# Whole-Genome Sequencing of Two Moroccan Mycobacterium tuberculosis Strains

**DOI:** 10.1128/MRA.00635-20

**Published:** 2021-01-07

**Authors:** M. Laamarti, N. El Mrimar, T. Alouane, S. Kartti, E. Belouad, F. Bssaibis, A. Zegmout, R. El Jaoudi, A. Maleb, A. Abid, N. El Hajjami, A. Lemnouer, S. Siah, L. Belyamani, M. Elouennass, A. Ibrahimi

**Affiliations:** a Biotechnology Lab (MedBiotech Center), Rabat Medical and Pharmacy School, University Mohammed V, Rabat, Morocco; b Department of Bacteriology, Mohammed V Military Teaching Hospital/Faculty of Medicine and Pharmacy, University Mohammed V, Rabat, Morocco; c Pneumology Department, Mohammed V Military Teaching Hospital/Faculty of Medicine and Pharmacy, University Mohammed V, Rabat, Morocco; d Department of Burns, Mohammed V Military Teaching Hospital/Faculty of Medicine and Pharmacy, University Mohammed V, Rabat, Morocco; e Laboratory of Microbiology, Mohammed VI University Hospital/Faculty of Medicine and Pharmacy, University Mohammed the First, Oujda, Morocco; f Emergency Department, Mohammed V Military Teaching Hospital/Faculty of Medicine and Pharmacy, University Mohammed V, Rabat, Morocco; Portland State University

## Abstract

Mycobacterium tuberculosis is known to cause pulmonary and extrapulmonary tuberculosis. In Morocco, the spread of multidrug-resistant (MDR) tuberculosis (TB) has become a major challenge. Here, we announce the draft genome sequences of two Mycobacterium tuberculosis strains, MTB1 and MTB2, isolated from patients with pulmonary tuberculosis in Morocco, to describe variants associated with drug resistance.

## ANNOUNCEMENT

Tuberculosis is an urgent public health problem in Morocco caused by Mycobacterium tuberculosis bacteria. Control of the bacteria has been recently complicated by the emergence of multidrug-resistant (MDR) strains showing resistance to the second-line treatment (rifampin, isoniazid) due to probable excessive antibiotic use (1–3). The identification of differences between MDR and sensitive Mycobacterium tuberculosis would improve the identification of the drug resistance site. Whole-genome sequencing using next-generation sequencing technologies is emerging as a rapid method for genetic characterization (4, 5). Thus, we provide the whole-genome sequencing data of two Mycobacterium tuberculosis strains, MTB1 and MTB2, with different resistance profiles.

Two Mycobacterium tuberculosis strains recovered from patients with pulmonary tuberculosis at the military hospital in Rabat, Morocco, and grown in Middlebrook 7H9 medium using the Bactec MGIT 320 system (Becton, Dickinson) were received for sequencing. DNA was purified using the Qiagen DNA extraction kit (QIAamp DNA minikit) following the kit protocol, and DNA libraries were prepared using the Nextera XT library preparation kit V3. Genomic DNA was sequenced using the Illumina MiSeq platform (San Diego, CA, USA) in paired-end (2 × 300-bp) format. Yields of 1,616,965 and 620,966 reads were preprocessed for quality checking using FastQC, further assembled using A5-miseq with default parameters (6) (the default settings include running Trimmomatic for read preprocessing) into 4,341,655-bp and 4,291,403-bp draft genome sequences, and divided into 242 (*N*_50_, 43,730 bp) and 238 (*N*_50_, 35,128 bp) contigs for MTB1 and MTB2, respectively.

The assemblies shared a GC content of 60% and 3 *arnT* operons. The genome annotation was performed using the NCBI annotation pipeline (7) and identified 4,265 and 4,422 coding DNA sequences (CDS) in MTB1 and MTB2, respectively.

Furthermore, reads were mapped to the h37rv reference genome using BWA (8). Variants were called using SAMtools (9) and annotated by SnpEff (10). The genome sequence displayed hot spot mutations in genes associated with resistance. MTB1 harbored mutations in *katG* (Ser315Thr) and *rpoB* (Ser450Leu) responsible for resistance to isoniazid and rifampin, respectively (11). No resistance-associated mutations were identified in the genes (*gyrA*, *gyrB*, and *rrs*) linked to second-line drugs (12). However, pyrazinamide resistance was due to a mutation in *pncA* (Gly97Asp) (13), which classifies MTB1 as pre-extensively drug resistant (pre-XDR), while MTB2 did not show any mutations associated with first- or second-line drug resistance in its genotype. This study represents an initial analysis of a Mycobacterium tuberculosis collection to highlight the resistance profile in Morocco.

### Data availability.

The genome sequences of Mycobacterium tuberculosis MTB1 and MTB2 have been deposited in DDBJ/ENA/GenBank under the accession numbers NARM00000000.1 and NARL00000000.1, respectively. The SRA accession numbers for MTB1 and MTB2 are SRR12031346 and SRR12031345, respectively.

## References

[1] Musser JM, Amin A, Ramaswamy S. 2000. Negligible genetic diversity of *Mycobacterium tuberculosis* host immune system protein targets: evidence of limited selective pressure. Genetics 155:7–16. 10790380 10.1093/genetics/155.1.7 PMC1461055

[2] Aghandous R, Zouhdi M, Zizi M, Errami M, Idrissi L. 1999. *Mycobacterium* resistance to antimycobacterial reagent. Therapie 54:623–625. 10667100

[3] El Baghdadi J, Lazraq R, Ibrahimy S, Bouayad Z, Guinet R, Benslimane A. 1997. Survey of primary drug resistance of *Mycobacterium tuberculosis* in Casablanca, Morocco. Int J Tuberc Lung Dis 1:309–313. 9432385

[4] Colman RE, Schupp JM, Hicks ND, Smith DE, Buchhagen JL, Valafar F, Crudu V, Romancenco E, Noroc E, Jackson L, Catanzaro DG, Rodwell TC, Catanzaro A, Keim P, Engelthaler DM. 2015. Detection of low-level mixed-population drug resistance in Mycobacterium tuberculosis using high fidelity amplicon sequencing. PLoS One 10:e0126626. doi:10.1371/journal.pone.0126626. 25970423 PMC4430321

[5] Colman RE, Mace A, Seifert M, Hetzel J, Mshaiel H, Suresh A, Lemmer D, Engelthaler DM, Catanzaro DG, Young AG, Denkinger CM, Rodwell TC. 2019. Whole-genome and targeted sequencing of drug-resistant Mycobacterium tuberculosis on the iSeq100 and MiSeq: a performance, ease-of-use, and cost evaluation. PLoS Med 16:e1002794. doi:10.1371/journal.pmed.1002794. 31039166 PMC6490892

[6] Coil D, Jospin G, Darling AE. 2015. A5-miseq: an updated pipeline to assemble microbial genomes from Illumina MiSeq data. Bioinformatics 31:587–589. doi:10.1093/bioinformatics/btu661. 25338718

[7] Tatusova T, DiCuccio M, Badretdin A, Chetvernin V, Nawrocki EP, Zaslavsky L, Lomsadze A, Pruitt KD, Borodovsky M, Ostell J. 2016. NCBI Prokaryotic Genome Annotation Pipeline. Nucleic Acids Res 44:6614–6624. doi:10.1093/nar/gkw569. 27342282 PMC5001611

[8] Li H, Durbin R. 2009. Fast and accurate short read alignment with Burrows-Wheeler transform. Bioinformatics 25:1754–1760. doi:10.1093/bioinformatics/btp324. 19451168 PMC2705234

[9] Li H. 2011. A statistical framework for SNP calling, mutation discovery, association mapping and population genetical parameter estimation from sequencing data. Bioinformatics 27:2987–2993. doi:10.1093/bioinformatics/btr509. 21903627 PMC3198575

[10] Cingolani P, Platts A, Wang LL, Coon M, Nguyen T, Wang L, Land SJ, Lu X, Ruden DM. 2012. A program for annotating and predicting the effects of single nucleotide polymorphisms, SnpEff: SNPs in the genome of Drosophila melanogaster strain w1118; iso-2; iso-3. Fly (Austin) 6:80–92. doi:10.4161/fly.19695. 22728672 PMC3679285

[11] Tracevska T, Jansone I, Broka L, Marga O, Baumanis V. 2002. Mutations in the rpoB and katG genes leading to drug resistance in Mycobacterium tuberculosis in Latvia. J Clin Microbiol 40:3789–3792. doi:10.1128/jcm.40.10.3789-3792.2002. 12354882 PMC130873

[12] Hu Y, Hoffner S, Wu L, Zhao Q, Jiang W, Xu B. 2013. Prevalence and genetic characterization of second-line drug-resistant and extensively drug-resistant Mycobacterium tuberculosis in rural China. Antimicrob Agents Chemother 57:3857–3863. doi:10.1128/AAC.00102-13. 23733477 PMC3719720

[13] Wu X, Lu W, Shao Y, Song H, Li G, Li Y, Zhu L, Chen C. 2019. pncA gene mutations in reporting pyrazinamide resistance among the MDR-TB suspects. Infect Genet Evol 72:147–150. doi:10.1016/j.meegid.2018.11.012. 30447296

